# Successful Use of Endoscopy for Transcervical Cannulation Procedures in the Goat

**DOI:** 10.1111/rda.12399

**Published:** 2014-09-15

**Authors:** A Colagross-Schouten, D Allison, L Brent, E Lissner

**Affiliations:** 1 BioConsult Veterinary GroupDavis, CA, USA; 2 Relief Veterinary ServicesDavis, CA, USA; 3 Parsemus FoundationBerkeley, CA, USA

## Abstract

Two methods for transcervical cannulation of the goat were evaluated during a contraception study in 15 adult female Nigerian dwarf and African pygmy goats. Twenty-four transcervical cannulation procedures were conducted in which seven females underwent the procedure 2–3 times. Initially, a rigid 4-mm stainless steel cannula and external light source were used in 19 procedures to introduce the contraceptive compound into the uterus. Placement of the cannula was directed by feel or depth assessment. Of seven females that were euthanized following this procedure, four evidenced complications including penetration of the cervix with the cannula and cervical damage. A 2-mm custom-made endoscope with a specially designed cannula was then used for the remaining five procedures. No complications were found. A single animal, that underwent the endoscopic procedure twice, was euthanized for study purposes and no abnormal findings of the reproductive tract were reported. The use of an endoscope resulted in better outcomes because the uterus could be visualized after traversing the cervix.

## Introduction

The cervix of small ruminants is a sphincter-like structure at the base of the uterus that is anatomically complicated consisting of a fibromuscular canal with multiple folds of tissue or rings. The size and shape of the cervix varies by breed and can change with the reproductive cycle, history and age of the female (Kershaw et al. 2005; Dayan et al. 2010).

The anatomy of the cervix makes it difficult to penetrate and gain access to the uterus, which may be required for assisted reproduction, experimental manipulations or diagnostic evaluation. Most research on transcervical techniques in ruminants has focused on artificial insemination (AI) and embryo transfer technologies in sheep. Deep penetration of the cervix required to obtain acceptable conception rates with frozen semen rarely occurs (King et al. 2004). Various methods and specialized instrumentation have been developed for transcervical access (Buckrell et al. 1994) but complications including damage to the cervix and puncture of the epithelium are common (Campbell et al. 1996) and lambing rates are usually lower than with laparoscopic AI (King et al. 2004). However, laparoscopic surgery raises welfare considerations and the requirement for specialized equipment and expertise.

Assisted reproduction technologies have been used in sheep and goats to improve genetics and reproductive output (Cognie et al. 2003). Transcervical embryo collection and transfer has been reported to be feasible in the goat (Pereira et al. 1998; Suyadi and Holz 2000; Lima-Verde et al. 2003) but less information is available on other transcervical procedures in this species. In this study, traversing the cervix was necessary to non-surgically place an experimental compound into the uterine body of mature female goats as part of a contraception study. Two methods were evaluated.

## Materials and Methods

### Subjects, housing and care

The subjects of this study were 15 female goats (*Capra aegagrus hircus*) aged 2–6 years and weighing 20.4–36.3 kg. Five were Nigerian dwarf and 10 were African pygmy goats, each with a sound reproductive history. Animals were housed in a large outdoor enclosure with shade structures and had access to water *ad libitum*, pelleted feed, grass hay and grazing opportunities. The goats received standard preventive care (routine weighing, deworming and hoof trimming).

### Experimental design

The goats participated in a contraception study, approved by the Institutional Animal Care and Use Committee that required the introduction of two different contraceptive compounds into the uterus. Fifteen goats were infused with one or both compounds via transcervical cannulation. Over the course of 65 weeks 24 transcervical cannulation infusions were performed: eight goats received one infusion, five goats received two infusions and two goats received three infusions. A cannula with specially designed endoscope was used to complete the second infusion in three females and a third infusion in two females, while all other infusions were completed with a cannula with external light source (see Cannulation procedures). The average time between the first and second infusion was 37.08 weeks (SD = 23.68) and between the second and third infusion was 56.57 weeks.

Eight subjects were later humanely euthanized: five as part of the research study, two for clinical reasons related to the procedure and one for an unrelated health issue. These females were given an intravenous overdose of pentobarbital and received a complete necropsy by a veterinary pathologist, which included a thorough gross exam of the reproductive tract. Tissues were fixed in formalin and routine paraffin light microscopic sections cut at 4–6 *μ*m and stained with haematoxylin and eosin and were examined with a Zeiss Axioskop light microscope at 20×–400× (Necropsy Services Group, Davis, CA, USA). The remaining seven subjects were adopted out after the study concluded.

### Oestrus determination

The goats were observed daily for signs of behavioural oestrus including vocalization, seeking out males, rubbing up against herd mates, tail flagging and restlessness. The vulva was also examined to determine the visual signs such as vulvar swelling and mucous discharge. Once animals were identified as being potentially in oestrus, a digital rectal exam was performed to assess the pliability of the cervix. If, based upon behavioural and physical signs, it appeared the doe was in late oestrus, further visual assessment of the cervix was performed. Does were given 0.05 mg/kg of xylazine IV to induce a mild sedative effect. After approximately 10 min, animals were gently restrained, usually across the lap of the seated handler. The perivulvar area was cleansed and a clear 1.9 cm speculum was introduced into the vaginal vault to visualize the cervix. A laryngoscope (AAS, LLC, Prescott, AZ, USA, laryngoscope with 13 mm Miller blade) or alternate light source was used to illuminate the cervical area. The colour and pliability of the cervix were assessed to assist in identifying late oestrus when the cervix was firm enough to retain the contraceptive compound. If the cervix appeared purplish in colour, and the os was slightly open with a small amount of mucus coming from it, an attempt at cannulation of the cervix was made.

### Cannulation procedures

Two methods were used to achieve access to the uterus: a cannula with external light source and a cannula with a specially designed endoscope. All equipment was sterilized and maintained aseptically during use.

**Figure 1:**
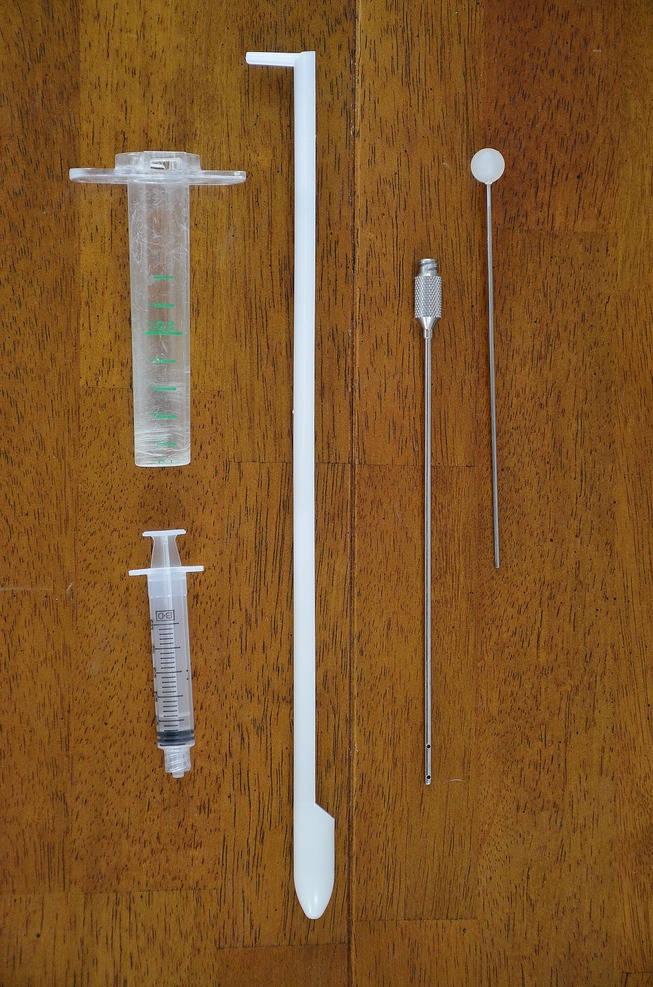
Cannula. From left to right: speculum with dosing syringe for flushing, vaginal introducer, cannula with stylet

The cannula is illustrated in Fig.1. The method was performed as follows: With a speculum in place, an external light source, typically a laryngoscope, was stabilized to have a clear view of the external os of the cervix. A rigid, stainless steel 4 mm cannula was introduced into the vaginal vault and placed against the os and, with gentle pressure, was slowly advanced through the opening. If moderate resistance was felt, the cannula was repositioned and one additional attempt was made. If the cannula was successfully passed through the external os of the cervix with only a slight resistance, advancement continued and the laryngoscope was withdrawn. Three or four ‘rings’ or cervical folds required navigating through before reaching the uterus.

**Figure 2:**
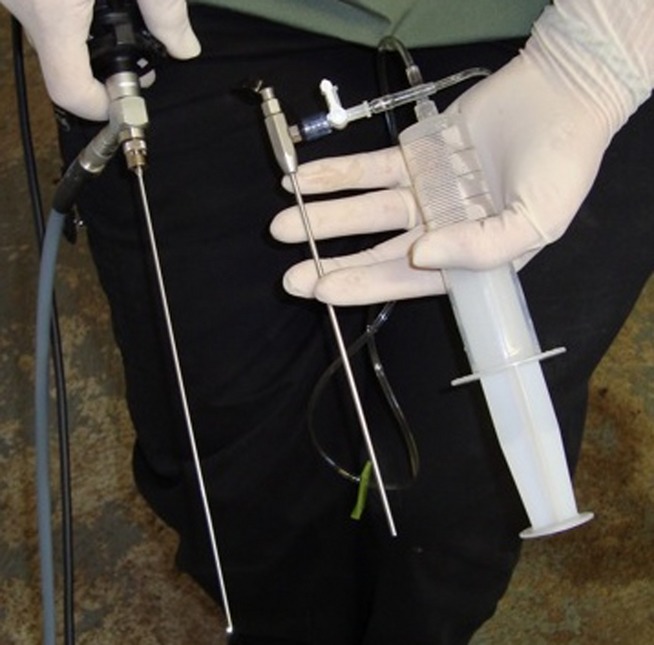
Specialized endoscope and cannula. From left to right: endoscope (fits within cannula), cannula with side administration port attached to syringe of saline for flushing

Alternatively, the cervix was visualized with an endoscope that was specially designed to fit within the cannula (Fig.2). The custom-made 2 mm, 24.3 cm and 0 degree endoscope had a universal eyepiece and was made by C-Link Micro Imaging, Inc. Los Angeles, CA USA. It came with a 2.4 mm, 17 cm custom-made dilator with irrigation port, LED light, 50 watt LED light source, fibre optic light guide and digital CCD camera. Using the endoscope, the cannula was continually advanced through the cervix with gentle pressure. Confirmation of placement was achieved when the wall of the uterine body could be seen and the endoscope could be advanced carefully to view the horns of the uterus.

Once placement within the uterus was confirmed by either cannulae advancement and depth assessment or visualization (endoscope), one of two contraceptive compounds was extruded through the cannula. The cannula was then carefully and slowly withdrawn. Goats were given a single dose of flunixin (∽50 mg/doe IM) immediately post-procedure for potential discomfort and reversed with atipamezole (50 mg IV). Animals were then released back to the herd and remained under the care of a veterinarian during the study.

## Results

**Figure 3:**
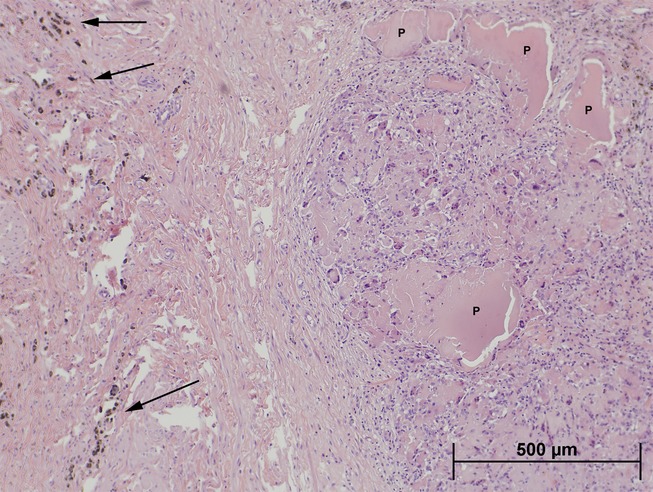
Histology of a section of the cervix from Animal 4. Contraceptive compound (P) has been deposited in the wall of the cervix and has produced a discrete foreign body inflammatory response containing multinucleated giant cells. Brown pigment (arrows) on the left side of the photograph (haemosiderin) indicates an area of resolved haemorrhage

The initial method to access the uterus of the subjects using a cannula and external light source was completed 19 times in the 15 females with four females undergoing the procedure twice. Two females were later euthanized due to complications. One had a ruptured uterus during delivery but no abnormal findings in the cervix. The other female who had received two transcervical procedures demonstrated signs of pain 24 h post-procedure and was euthanized. She appeared to have penetration of the cervix with abdominal deposition of contraceptive compound, which had been encapsulated into multiple foci of inflammation with peritoneal effusion. One female was euthanized due to an unrelated health issue and no abnormal findings of the reproductive tract were identified. Four other females did not have clinical signs but were euthanized and necropsied for study purposes. In one female, the cervix had been penetrated and product extravasated into the surrounding serosa with a resultant giant cell foreign body response. The second female presented with multiple adhesions between the cervix and other internal tissues and a 4-cm cervical swelling. The third doe had regional erosion of the cervical mucosa with underlying chronic inflammation (Fig.3). The fourth female had no abnormal findings of the reproductive tract despite having undergone two transcervical procedures (Table1).

**Table 1:** Summary of procedures and outcome for each subject

Number	Infusion 1	Infusion 2	Infusion 3	Outcome
1	External light source	Endoscope		Adopted
2	External light source			Euthanized for ruptured uterus during delivery. No abnormal findings
3	External light source			Euthanized for study. Cervix penetrated and product extravasated
4	External light source			Euthanized for study. Adhesions and cervical swelling
5	External light source	Endoscope	Endoscope	Euthanized for study. No abnormal findings
6	External light source			Adopted
7	External light source			Euthanized for unrelated causes. No abnormal findings
8	External light source	External light source		Adopted
9	External light source			Adopted
10	External light source			Adopted
11	External light source	External light source	Endoscope	Adopted
12	External light source			Euthanized for study. Erosion of cervical mucosa and inflammation
13	External light source	Endoscope		Adopted
14	External light source	External light source		Euthanized due to pain following procedure. Penetration of uterus and deposition of contraceptive compound into abdomen
15	External light source	External light source		Euthanized for study. No abnormal findings

In an attempt to eliminate adverse outcomes, the endoscope was then used in five procedures on four females. No complications were found. A single animal that had undergone two endoscopic procedures was euthanized for study purposes and no abnormal findings of the reproductive tract were reported.

## Discussion

**Figure 4:**
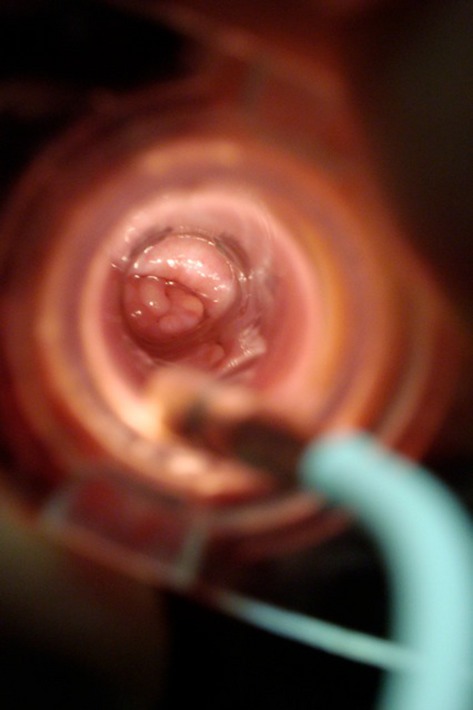
Cervix visualized with clear speculum and external light source

Traversing the cervix of small ruminants is difficult due to the variation and structure of the cervix. Thus, transcervical procedures are uncommon, necessitating surgical access for experimental, clinical and most reproductive technologies. In goats, some studies reported that successful transcervical embryo collection or AI were conducted (Pereira et al. 1998; Sohnrey and Holtz 2005; Fonseca et al. 2013), but others reported that only 61% of goat cervices were penetrable (Lima-Verde et al. 2003). In our study, transcervical placement of an experimental contraceptive compound was hampered by the difficulty in accessing the uterus and resulted in adverse outcomes for some of the subjects. We found that use of an endoscope resulted in better outcomes because the uterus could be visualized after crossing the cervix (Fig.4), confirming correct and atraumatic placement, whereas using a cannula and external light source necessitated placing the compound based only on depth assessment and feel during cannula advancement.

A custom endoscope can be expensive, but the value in successfully completing transcervical cannulation procedures may be worth the cost of the equipment when used frequently, such as for assisted reproduction programmes. This method also enhanced animal welfare by alleviating the need for surgery.
